# Unweighted regression models perform better than weighted regression techniques for respondent-driven sampling data: results from a simulation study

**DOI:** 10.1186/s12874-019-0842-5

**Published:** 2019-10-29

**Authors:** Lisa Avery, Nooshin Rotondi, Constance McKnight, Michelle Firestone, Janet Smylie, Michael Rotondi

**Affiliations:** 1 York University, 4700 Keele St, Toronto, ON M3J 1P3 Canada; 2 University of Otago, 362 Leith St, North Dunedin, Dunedin, 9016 New Zealand; 3 Well Living House, Centre for Urban Health Solutions, St. Michael’s Hospital, 30 Bond St, Toronto, ON M5B 1W8 Canada; 4 Faculty of Health Sciences, Ontario Tech University, 2000 Simcoe St. North, Oshawa, ON L1H 7K4 Canada; 5 De dwa da dehs nye>s Aboriginal Health Centre, 678 Main St E, Hamilton, ON L8M 1K2 Canada

## Abstract

**Background:**

It is unclear whether weighted or unweighted regression is preferred in the analysis of data derived from respondent driven sampling. Our objective was to evaluate the validity of various regression models, with and without weights and with various controls for clustering in the estimation of the risk of group membership from data collected using respondent-driven sampling (RDS).

**Methods:**

Twelve networked populations, with varying levels of homophily and prevalence, based on a known distribution of a continuous predictor were simulated using 1000 RDS samples from each population. Weighted and unweighted binomial and Poisson general linear models, with and without various clustering controls and standard error adjustments were modelled for each sample and evaluated with respect to validity, bias and coverage rate. Population prevalence was also estimated.

**Results:**

In the regression analysis, the unweighted log-link (Poisson) models maintained the nominal type-I error rate across all populations. Bias was substantial and type-I error rates unacceptably high for weighted binomial regression. Coverage rates for the estimation of prevalence were highest using RDS-weighted logistic regression, except at low prevalence (10%) where unweighted models are recommended.

**Conclusions:**

Caution is warranted when undertaking regression analysis of RDS data. Even when reported degree is accurate, low reported degree can unduly influence regression estimates. Unweighted Poisson regression is therefore recommended.

## Background

Respondent-driven sampling (RDS) was developed by Heckathorn [1] as an improvement on snowball-type sampling for measuring disease prevalence in ‘hidden’ populations, that is, those that are difficult to reach because they lack a sampling frame. Groups commonly studied with RDS include men who have sex with men, sex workers and drug users [2–4]. The intricacies of RDS are described elsewhere [1, 5–7] so we provide only a brief outline here. Researchers recruit an initial group from the target population, called ‘seeds’. Each seed is tasked with recruiting members from their personal network who are also members of the target population; these recruited participants then become recruiters themselves and sampling continues until a pre-specified condition is met, typically when the target sample size is reached. Usually, participants are incentivized to participant in the recruitment chains by receiving payment both for participating and for recruiting others into the study. Recruitment is tracked using coupons so that participants can be traced along the recruitment chains. Participants are also asked about the size of their personal networks with respect to the population of interest. For example, in a study of HIV prevalence among injection drug users in a city, participants may be asked: “How many other people who inject drugs in [city] do you spend time with?”. The resulting RDS data differs in two important aspects from data obtained through simple random samples. First, sampling is not random, some participants are more likely to be selected than others and this likelihood is a function of how well-connected they are. Second, the observations are not independent as the data may be clustered within recruiters or seeds.

Clustering occurs if there is homophily in the population; if people are more likely to be connected to others with a shared trait; although it can also refer to network communities as outlined by Rocha et al. [8]. In this paper, we consider clustering within a single community and therefore driven by homophily. Heckathorn showed that, if the recruitment chains are long enough, under certain (reasonable) assumptions the RDS-derived data can be analysed in such a way as to produce asymptotically unbiased population estimates of disease prevalence [7]. The utility of RDS-specific prevalence estimates has been studied using simulation by Spiller et al. [9] and Baraff, McCormick and Raftery [10] who examined the variability of RDS prevalence estimates and recommended RDS-specific techniques instead of naive sample prevalence estimates. However, McCreesh et al. [11] cautioned that in estimates of prevalence, RDS-adjusted techniques often produced confidence intervals that excluded the population value. Until recently, the focus of most studies using RDS has been to quantify disease prevalence, but as RDS becomes more popular, regression analyses of these data are also becoming common.

Although regression analysis of RDS data is frequently undertaken, the best method for accommodating correlation between participants (clustering) and the non-random sampling of recruits remains unknown. Carballo-Diéguez et al. [12] noted in 2011 that “the pace of development of statistical analysis methods for RDS-collected data has been slower than the explosion of implementation of RDS as a recruitment tool”. Several authors have recently observed that regression techniques in particular for RDS samples are not well established [4, 13, 14]. Yet their use continues to increase; a search of PubMed for the terms ‘respondent driven sampling’ and ‘regression’ over the years 1997 to 2017 indicated that the first RDS paper to use regression techniques was published in 2004, by 2017 there were 59 papers. While many authors do not specifically address the difficulties in performing regression on RDS data some acknowledge the limitations and perform unadjusted analysis [4, 13]. Several authors used weighted regression [14–18], which assumes that network size is accurately reported and without further adjustment still assumes independence between participants; or included weights as covariates [17, 18]. At least one study mitigated the influence of extreme responders to the network question with the ‘pull-in’ feature of the RDSAT software [19] which re-assigns extreme values to ones more aligned with the sample [20]. Fewer authors have attempted to control for clustering; Lima et al. attempted to control for homophily (related to clustering) by incorporating the outcome value of the recruiter as an independent variable [21] and Schwartz et al. used robust Poisson regression ‘accounting for clustering’ of participants within the same seed [13]. We found only one study which used both weighted regression and controlled for clustering; those authors used weighted regression and modelled dependence among observations with two methods and found similar results with both [22]. Treatment of clustering is the thornier of the two statistical issues with RDS regression, because clusters, if they exist, may be difficult to identify. The main clustering unit may be at the level of the seed, which would produce a few, large clusters, or it may be approximated by an auto-regressive structure in which participants are dependent on their immediate recruiter, but largely independent of those further up the recruitment chain. The covariance structure proposed by Wilhelm [23] in which correlation decreases with successive waves may provide a useful middle ground. Added to these conceptual questions are statistical concerns with clustered data. Hubbard at al [24]. note that when generalised estimating equations (GEE) are used, estimates can be inaccurate if the number of clusters is small, so treating initial seeds as clustering units can be problematic. Another study with mixed cluster sizes found that failure to adjust for clustering would have led to incorrect conclusions [25]. There are a multitude of methods available to account for both unequal sampling probabilities and clustering, but little work has been undertaken to determine the most appropriate regression methods for use with RDS data.

### Motivating example

The Our Health Counts (OHC) Hamilton study was a community-based participatory research project with the aim of establishing a baseline health database for an urban Indigenous population living in Ontario. Respondent-driven sampling was appropriate for this population because of the inter-connectedness of the population and the lack of a suitable sampling frame. Based on census estimates, the population is comprised of approximately 10,000 individuals, 500 of whom were sampled in the OHC study. Commonly reported network sizes are 10, 20, 50 and 100, the median network size was 20, with mean 46.5. The top decile of participants reported network sizes in excess of 100 people. The distribution of reported network size for the OHC Hamilton study is illustrated in the Additional file 1: Figure S1.

The objective of this simulation study was to evaluate the validity and accuracy of several regression models for estimating the risk of a binary outcome from a continuous predictor from an RDS sample and specifically, to assess performance with varying levels of outcome prevalence and homophily.

## Methods

**Fig. 1:**
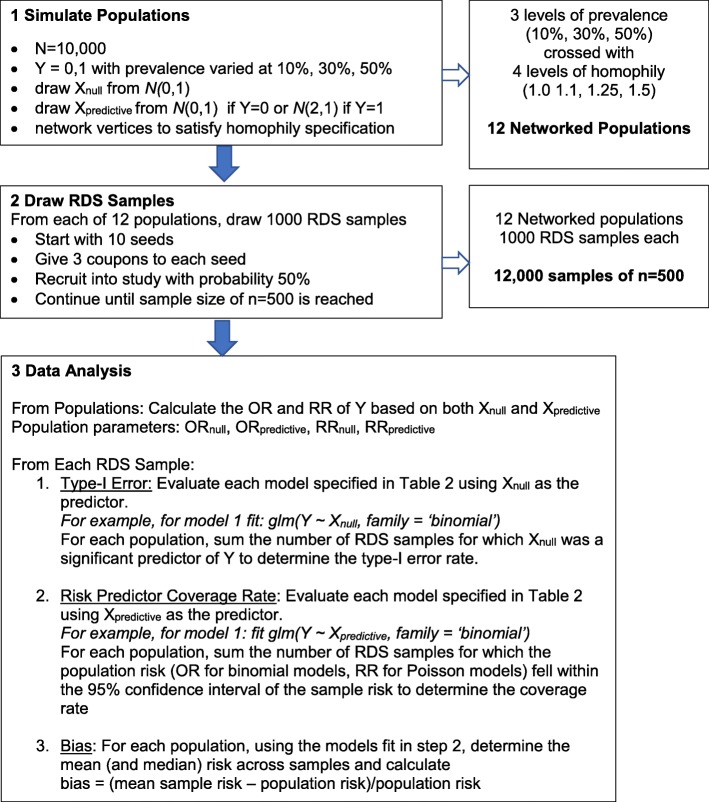
Illustration of study workflow

We conducted a simulation study in which networked populations were created, 1000 samples were drawn from these simulated populations using RDS and the samples were analyzed to evaluate the performance of various regression models. Our methods are explained in detail below and a visual overview of the workflow is shown in Fig. 1.

### Data simulation

#### Population generation

Populations of 10,000 networked individuals were simulated. Each individual was assigned four traits: a binary trait indicating group membership (G1: Y=1 or G2: Y=0) with probability of G1 = *π*, a continuous predictor (*X*_*predict*_) such that *X*_*predict*_ ∼ *N*(2, 1) for G1 and *X*_*predict*_ ∼ *N*(0, 1) for G2, a second continuous predictor, *X*_*NULL*_ ∼ *N*(0, 1) for all individuals (to evaluate the type-I error rate) and a network degree, *d*_*i*_, specifying the number of connections with other members of the population. The proportion of the population in G1 (*π*), known as the outcome prevalence henceforth, was varied at 10, 30 and 50%; this would normally refer to disease prevalence in RDS studies. Relative activity (*ω*), the ratio of the average reported network size in G2 relative to G1, was fixed at 1 for all populations. Population homophily (Hx), the proportion of within group to between group links in the population, was defined as follows:

$$ Hx=2\pi \left(1-\pi \right)\left(\frac{T_{ii}}{T_{ij}}+1\right) $$

where *T*_*ii*_ and *T*_*ij*_ are the number of within group and between group ties, respectively. Homophily was varied at 1.0, 1.1, 1.25 and 1.5. Each level of homophily was crossed with each level of population prevalence to produce 12 simulated networked populations consistent with the range of outcomes and homophily levels that were observed in the OHC Hamilton study.

Network degree was drawn from the distributions shown in the Additional file 2: Figure S2, which is comprised of a series of binomial distributions designed to mimic the modes reported in the OHC Hamilton study. The generating distribution for this simulation study had similar properties to the OHC Hamilton sample, with overall median degree 20 and mean degree 47.5. However, in the OHC data degrees were often reported as multiples of 5, 10 or 100, which did not occur in our simulated samples due to the exact knowledge of degrees from the simulated populations.

#### Secondary populations

As a secondary analysis to determine if a correlation between network degree and outcome affected our results we simulated eight additional populations. Outcome prevalence was fixed at 10%, homophily was varied at 1.25 and 1.5. Four different levels of outcome-degree correlation were modelled: 1. Extreme positive correlation, where the members of G1 were assigned the highest network degrees. 2. Moderate positive correlation, where, beginning with the top decile of network size 50% more individual were assigned to G1 than would be expected, and this process was repeated with successive deciles until 10% of the population had been assigned to G1. 3. Moderate negative correlation, as with #2 but assignment to G1 began with the lowest degree decile. 4. Extreme negative correlation, as with #1, but assignment to G1 was allocated to subjects with the lowest network degree.

#### RDS sampling

**Fig. 2:**
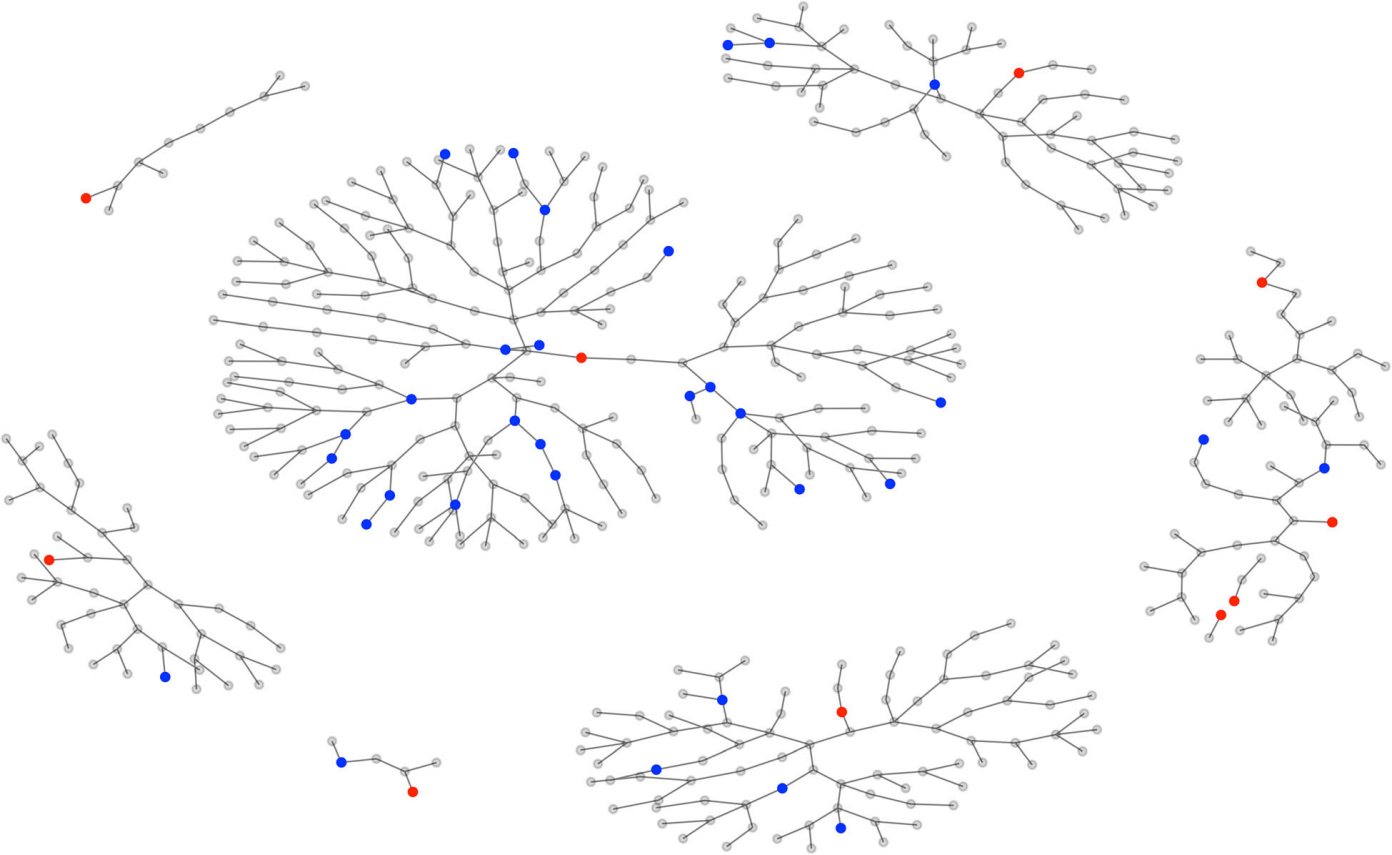
Simulated RDS Sample from a population with homophily of 1.5 and population prevalence of 0 10%. Red dots indicate the seeds and blue dots are members of Group 1

From each population, 1000 RDS samples were drawn as follows. Ten seeds were randomly drawn. Non-response was set to 50% in each group, to mimic real world conditions and to extend the recruitment chains. Three coupons were ‘given’ to each respondent and sampling continued, wave by wave, until the desired sample size of 500 was reached. Although sampling with replacement is an assumption of the random-walk model on which RDS methods are based [5] repeat recruitment was not allowed in this study, as is the case in real-world applications. Figure 2 is a graph of a single RDS sample from a population with *π* =10% and Hx = 1.5; members of G1 are shown as blue dots, seeds are shown as red dots.

Data simulation was performed by modifying the *RDS Release* [23] code in the R statistical language [26]; the networked populations and samples are available on github.

### Data analysis

#### Population parameters

Odds ratio and relative risk of membership in G1, for each unit increase in the random variable (*X*_*predict*_), were calculated for each population using generalized linear models with binary and logistic links respectively. For calculation of the population parameters there is no need to adjust for clustering or unequal sampling probability so unadjusted analyses were performed using the glm function in R [26]. To ensure that the RDS sampling did indeed sample participants proportional to their network degree we counted the number of RDS samples each participant appeared in (their sampling frequency) and looked at the correlation between sampling frequency and network degree across all populations.

#### Model fitting

Three main approaches were used to model the simulated sample data. Standard logistic regression models (GLM), in which the log-odds of belong in G1 (vs G2) is modelled as a linear function of the continuous predictor (X), were fit using both the surveylogistic function in SAS [27] and the glm function in R [26]. Generalized linear mixed models (GLMM) are an extension of GLM in which correlation in the sample, caused by clustering within seeds and recruiters can be modelled with random effects. These models were fit using the glimmix procedure in SAS and the glmer [28] and glmmPQL [29] functions in R. Finally, generalized estimating equations (GEE) were modelled, using the geeglm function in R [30] and the glimmix function in SAS. These models are often referred to as population-average models because the fixed-effects estimates represent population average across all values of the random effects, which are not separately estimated, but described by an estimated covariance matrix. To compensate for mis-specification of the covariance structure, GEE estimates can be corrected with variance adjustments. A more thorough explanation of these different models is provided by Rao et al. [25].

In addition to binomial regression with logit link, a subset of models was also fit using Poisson regression with loglinear link. In the interest of parsimony, not every possible model combination was explored, but instead we focused on models reported in the literature and models we thought may be useful; thus a total of 31 models were tested. A complete summary of each of the models is included in the results. Unless otherwise specified, program defaults were used; ie glimmix procedures used the default pseudo-likelihood residual based ‘RSPL’ method. Seeds were excluded from the analyses. Every model was evaluated twice for each sample, once using *X*_*NULL*_ to evaluate validity and once using *X*_*predict*_ to evaluate the coverage rate for the predictive continuous variable. An explanation of model specifications follows.

#### Weighting

Unequal sampling probability is one of the main differences between RDS samples and simple random samples. In this simulation study we had the advantage of knowing precisely the degree to which each participant was connected to others in the population. Standard weighted regression was undertaken using the Volz-Heckathorn (RDS-II) weights [31] from the RDS package [32]. These are inverse probability weights, based on the reported network degree (assumed to be a proxy for the sampling probability) and defined as:

$$ {w}_i=\frac{1}{d_i}\frac{\sum_{i=1}^N\frac{1}{d_i}}{N} $$

where *d*_*i*_ is the reported network size.

#### Clustering

In RDS data participants are clustered within their immediate recruiter and within the recruitment chains, defined by the original seeds. Several different approaches were used to account for this clustering. For glm models, the outcome status of each participant’s recruiter was included as a model covariate, as per Lima et al. [21] (models 3–4, 26–27). For the surveylogistic models fit in SAS (models 9, 10) the *strata* and *class* commands were used to define observations within recruiters within seeds. Several methods were used for the GLMM models: the glmer function was used to model unstructured covariance within seeds (models 11–12, 28–29), glimmix was used to model first-order auto regressive correlation along recruitment chains (models 13) and immediate recruiters as the clustering unit, with exchangeable correlation structure (model 14), glmmPQL in the glmm package [33] was used to model a declining correlation structure as described in Beckett et al. [22], in which the correlation decreases with increased distance along the recruitment trees (model 15). Finally, in the GEE models, geeglm from the geepack package [30] was used to fit an independent working covariance structure within recruiters (models 16–17, 30–31), and glimmix was used to fit auto-regression correlation along recruitment lines (model 18) and exchangeable working correlation structures within recruiter (models 19–23). In models with no clustering unit specified in Table 2 the clustering within recruitment chains was ignored (models 1–2, 5–8, 24–25).

#### Variance adjustments

To reduce the impact of a mis-specified covariance structure, various adjustments (known as bias-corrected sandwich estimators) were used. The classical robust sandwich estimator, FIRORES, FIROEEQ and the Morel, Bokossa and Neerchal (MBN) were all tested; these estimators are described in detail elsewhere [25, 34, 35]. The variance adjustments applied to each model are detailed in Table 2, most models were unadjusted.

#### Evaluating fitted models

Observed type-I error rate, parameter coverage rate and bias were assessed for each model. Parameter coverage rate was defined as the proportion of simulations in which the 95% confidence interval of the risk parameter contained the true population value. This approach was used in preference to a calculation of power to better evaluate the ability of our regression models to discriminate between distinct groups in a confidence interval-based framework. Type I error was assessed using the models in which the independent variable was *X*_*NULL*_, and coverage rate was assessed with an independent variable of *X*_*predict*_. To compare models estimating odds ratios with those estimating relative risk, the bias of the risk estimates was considered, defined as $bias=\frac{mean\left(\hat{\theta}\right)-\theta }{\theta }$, where *θ* was the odds ratio for logit link models and the relative risk for Poisson models. Bias was calculated with respect to both the mean and median. The type-I error rate was calculated by fitting each model a second time, replacing the continuous predictor *X* with the second predictor, *X*_*NULL*_ and calculating the proportion of simulations with a *p*-value ≤ 0.05. Overall error, coverage rate and bias were calculated across all 12 simulated populations. To evaluate the predictive ability of the models, model accuracy was calculated for those models with observed error rate ≤0.05 *and* observed coverage rate ≥0.95. Accuracy was defined as the proportion of subjects whose disease status was accurately predicted, specifically:

$$ Accuracy=\frac{1}{N}\sum \limits_{i=1}^NI\left({p}_i\ge 0.5\  and\ {g}_i=1\right)+I\left({p}_i<0.5\  and\ {g}_i=0\right) $$

Because some models required knowledge of the outcome status of a participant’s recruiter (models 3, 4, 26, 27) and this information is not available for seeds, seeds were not included in the regression analysis.

For the secondary analysis on the correlated outcomes the type I error rate was focused on four models: unweighted binomial and poisson generalized linear models and weighted binomial and poisson generalized linear models (models 1, 2, 24, 25 from Table 2).

#### Outcome prevalence

To confirm that RDS-II weights were the appropriate observation weights, outcome prevalence was calculated for each sample, within each population. Using R and the RDS package [32] the naïve, RDS-I, RDS-II prevalence estimates were calculated. In SAS [27] the surveylogistic procedure was used to calculate the unweighted and observation-weighted prevalence, with and without the Morel standard error adjustment.

## Results

### Population parameters

Table 1 describes the 12 simulated populations. All populations have similar network and random variable characteristics, and are in line with target values. Mean network degree, number of waves, and number of recruits per seed are consistent across populations. In these populations, with relatively high outcome proportion, the odds ratio is a poor estimate of the relative risk.

**Table 1:** Population and mean sample characteristics for each simulated population

Population	Population characteristics				Mean sample characteristics			Sampling correlation^a^
	Prevalence	Homophily	Odds ratio	Relative risk	Degree	Number of waves	Recruits per seed	
1	10%	1.00	7.59	2.86	44.4	8.4	57.5	0.899
2	10%	1.10	7.65	2.88	43.5	8.3	57.2	0.895
3	10%	1.25	7.22	2.84	44.2	8.4	57.0	0.900
4	10%	1.50	6.93	2.85	43.7	8.3	56.9	0.896
5	30%	1.00	7.47	2.05	43.8	8.1	55.9	0.896
6	30%	1.10	7.56	2.05	43.4	8.1	55.6	0.891
7	30%	1.25	7.47	2.05	44.4	8.2	55.9	0.894
8	30%	1.50	7.59	2.06	44.2	8.2	56.3	0.894
9	50%	1.00	7.47	1.68	43.6	8.2	55.6	0.890
10	50%	1.10	7.55	1.68	43.5	8.1	55.6	0.890
11	50%	1.25	7.50	1.69	44.2	8.2	55.3	0.892
12	50%	1.50	7.51	1.69	44.0	8.2	55.9	0.893

^a^Correlation between network degree and sampling frequency

### Regression model performance

Model performance assessed across all populations is presented in Table 2. Results for individual populations are presented in the Additional files  5, 6, 7, 8 and 9.

**Table 2:** Summary of regression model performance across all populations

	Model	Weight	Clusters	Ψ	SE Adj.	Error	Coverage	Bias (mean %)	Bias (median %)	Accuracy (%)
Logistic Regression										
Generalised Linear Models										
glm(R)	1	–				0.04	0.954	2.07	−1.63	88.1
	2	RDS-II				0.55	0.442	20.89	8.51	
	3	–	R-y			0.04	0.955	3.35	−0.48	88.6
	4	RDS-II	R-y			0.55	0.443	25.56	11.57	
surveylogistic (SAS)	5	–				0.05	0.952	2.07	−1.63	88.1
	6	RDS-II				0.07	0.903	20.88	8.51	
	7	–			Morel	0.05	0.953	2.07	−1.63	88.1
	8	RDS-II			Morel	0.07	0.904	20.88	8.51	
	9	RDS-II	RwS			0.07	0.903	20.88	8.51	
	10	RDS-II	RwS		Morel	0.07	0.904	20.88	8.51	
Generalised Linear Mixed Models										
glmer(R)	11	–	S	U		0.05	0.954	3.48	−0.46	88.1
	12	RDS-II	S	U		0.55	0.402	44.55	26.73	
glimmix (SAS)	13	–	S	AR		0.04	0.955	3.45	−0.34	88.1
glimmix (SAS)	14	–	R	CS		0.04	0.957	2.4	−1.19	88.1
glmmPQL(R)	15	–	S	DC		0.04	0.865	−0.86	−6.34	
Generalised Estimating Equations										
geeglm(R)	16	–	R	I	Classical	0.13	0.952	2.07	−1.63	
	17	RDS-II	R	I	Classical	0.16	0.902	20.89	8.51	
glimmix (SAS)	18	–	S	AR		0.04	0.939	1.85	−1.69	
	19	–	R	CS		0.04	0.937	2.52	−1.75	
	20	–	R	CS	Classical	0.05	0.948	2.52	−1.75	
	21	–	R	CS	FIRORES	0.05	0.950	2.52	−1.75	88.1
	22	–	R	CS	FIROEEQ	0.05	0.951	2.52	−1.75	88.1
	23	–	R	CS	MBN	0.05	0.950	2.52	−1.75	
Poisson Regression										
Generalised Linear Models										
glm(R)	24	–				0.02	0.962	4.81	4.15	86
glm(R)	25	RDS-II				0.49	0.457	9.48	8.23	
glm(R)	26	–	R-y			0.02	0.964	3.06	2.44	86.3
glm(R)	27	RDS-II	R-y			0.47	0.493	7.74	6.46	
Generalised Linear Mixed Models										
glmer(R)	28	–	S	U		0.02	0.963	4.92	4.27	86
	29	RDS-II	S	U		0.47	0.431	11.71	10.42	
Generalised Estimating Equations										
geeglm(R)	30	–	R	I	Classical	0.13	0.859	4.81	4.15	
	31	RDS-II	R	I	Classical	0.17	0.781	9.48	8.23	

*R-y* recruiter outcome as covariate, *S* Seeds, *R* recruiter, *RwS* recruiter within seed

### Type-I error rate

Of the 31 models tested, 13 had consistently inflated error rates (> 0.05) across every populations: all 12 weighted regression models as well as the two GEE models fit with independent working correlation structure using the geeglm function (models 16, 30). Of the 17 remaining models, type-I error was generally close to the nominal rate of 0.05, but notably lower for the Poisson GLM models, which were the only models with observed error rate ≤ 0.05 for each and every population. Error rate was often inflated for the population with outcome prevalence of 50% and the largest degree of homophily for binomial models, but not for Poisson models which recorded lower than expected error rates in this population. The observed type-I error rate across 1000 RDS samples for each simulated population is included in Additional file 5: Table S1.

### Risk parameter coverage rates

Risk parameter coverage rates were calculated as the proportion of samples in which the 95% confidence interval of the risk estimate (the unit increase in risk attributable to *X*_*predict*_) included the true population parameter. Models using regression weights had poor coverage. The GLMM model fit with the declining correlation structure suggested by Beckett et al. [22] exhibited low parameter coverage rate, despite an acceptable error rate, due to underestimation of the parameter variance. This was also the only model for which there were any problems with convergence; 1–13% of the simulated RDS samples did not result in sensible standard errors (reported as either infinite or zero). In general, the GEE models had slightly lower than expected coverage rates (models 16–23,30,21). However, the FIRORES and FIROEEQ adjustments to the standard error resulted in coverage rates in the expected range. Additional file 6: Table S2 reports coverage rates across 1000 RDS samples for each simulated population.

### Bias

Additional file 7: Tables S3 and Additional file 8: Table S4 describe the relative bias of the risk estimates for each model. Bias with respect to the median was substantially lower than with respect to the mean, indicating that some samples had very large risk estimates. The Poisson regression models had similar bias whether respect to the mean or the median and were of larger magnitude than the corresponding Binomial model.

### Accuracy

**Fig. 3:**
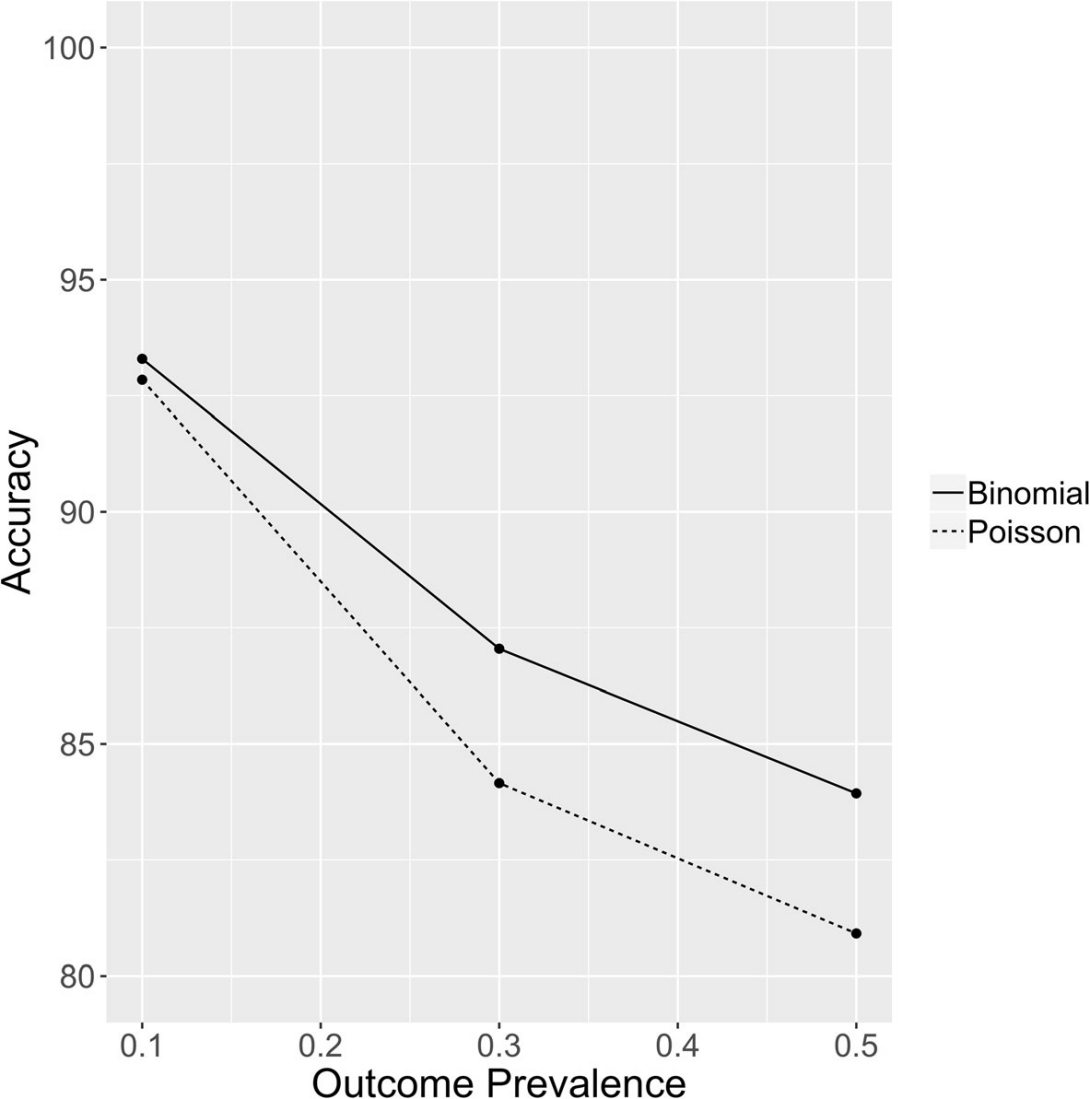
Prediction accuracy of the unweighted Binomial (model 1) and Poisson (model 24) for the populations with homophily of 1

Predictive accuracy was largely independent of the level of population homophily, but decreased with increased outcome prevalence. The unweighted binomial model with participants’ recruiter’s outcome variable included as a model predictor had the best accuracy, closely followed by the regular unweighted binomial model. Accuracy of the Poisson regression models decreased more quickly than that of the Binomial models for increased outcome prevalence, as shown in Fig. 3. Additional file 9: Table S5 details the accuracy across all populations.

### Disease prevalence

Table 3 reports the mean and standard deviation of the observed sample prevalence estimates across populations, along with the coverage rate for the naïve, RDS-II and surveylogistic procedure. All estimators tended to underestimate the true prevalence, with similar mean prevalence estimates across estimators. None of the estimators had coverage at the nominal rate. The best coverage was achieved using the weighted surveylogistic procedure, except at low prevalence (10%), where the unweighted procedure was superior. The Morel adjustment to the estimation of variance produced results identical to the default degrees of freedom adjustment used by SAS, to two decimal places and is not reported.

**Table 3:** Outcome prevalence estimates using various estimators across populations

Homophily:	Outcome prevalence 10%				Outcome prevalence 30%				Outcome prevalence 50%			
	1.00	1.10	1.25	1.50	1.00	1.10	1.25	1.50	1.00	1.10	1.25	1.50
Mean outcome prevalence												
naïve	0.09	0.09	0.09	0.09	0.27	0.27	0.27	0.27	0.47	0.47	0.47	0.46
RDS-I	0.08	0.08	0.08	0.08	0.27	0.26	0.26	0.26	0.47	0.47	0.46	0.46
RDS-II	0.08	0.08	0.08	0.08	0.27	0.26	0.26	0.26	0.47	0.47	0.46	0.46
surveylogistic models												
unweighted	0.09	0.09	0.09	0.09	0.27	0.27	0.27	0.27	0.47	0.47	0.47	0.46
weighted (RDS-II)	0.08	0.08	0.08	0.08	0.27	0.26	0.26	0.26	0.47	0.46	0.46	0.45
Mean SD of outcome prevalence												
naive	0.01	0.01	0.01	0.02	0.02	0.02	0.02	0.03	0.02	0.02	0.03	0.03
RDS-I	0.02	0.02	0.02	0.03	0.04	0.04	0.04	0.04	0.04	0.05	0.05	0.05
RDS-II	0.02	0.02	0.02	0.03	0.04	0.04	0.04	0.05	0.04	0.05	0.05	0.05
surveylogistic models												
unweighted	0.01	0.01	0.01	0.02	0.02	0.02	0.02	0.03	0.02	0.02	0.03	0.03
weighted (RDS-II)	0.02	0.02	0.02	0.03	0.04	0.04	0.04	0.04	0.04	0.05	0.05	0.05
Estimator coverage rates												
naive	0.845	0.827	0.802	0.708	0.646	0.740	0.620	0.642	0.742	0.687	0.634	0.551
RDS-I	0.545	0.554	0.548	0.578	0.572	0.512	0.524	0.501	0.627	0.610	0.569	0.511
RDS-II	0.772	0.776	0.766	0.749	0.799	0.761	0.744	0.723	0.839	0.831	0.791	0.741
surveylogistic models												
unweighted	0.916	0.900	0.875	0.784	0.657	0.745	0.611	0.645	0.747	0.684	0.644	0.544
weighted (RDS-II)	0.828	0.819	0.799	0.769	0.825	0.779	0.778	0.753	0.862	0.835	0.819	0.756

### Secondary analysis: correlated degree and outcome

Table 4 reports the type I error rate for the secondary populations. Type I error was affected by the correlation between the outcome and network degree for weighted, but not unweighted analyses. In the populations with extreme positive correlation, where those in G1 had the highest network degrees (and therefore the lowest RDS-II weights) the observed error rate was < 0.01, for the other populations the error rate for the weighted regression is well in excess of the nominal rate of 0.05. Error rates for the unweighted analyses are similar to those reported in the uncorrelated samples and near the nominal level.

**Table 4:** Type I error rate of unweighted and weighted regression models for populations with correlation between outcome and network degree

Secondary analysis population		Binomial regression		Poisson regression	
Correlation of degree and outcome		Unweighted	Weighted	Unweighted	Weighted
Population homophily = 1.25					
1	extreme negative (*ρ* = −0.133)	0.043	0.548	0.037	0.455
2	extreme positive (*ρ* = 0.534)	0.048	0.003	0.037	0.003
3	moderate negative (*ρ* = −0.092)	0.062	0.498	0.049	0.445
4	moderate positive (*ρ* = 0.059)	0.046	0.241	0.032	0.229
Population homophily = 1.50					
5	extreme negative (*ρ* = −0.132)	0.037	0.529	0.029	0.412
6	extreme positive (*ρ* = 0.534)	0.054	0.006	0.043	0.006
7	moderate negative (*ρ* = −0.093)	0.037	0.459	0.025	0.418
8	moderate positive (*ρ* = 0.060)	0.024	0.186	0.020	0.175

## Discussion

Using simulated data, with network degree modelled after RDS data collected from an urban Indigenous population, a dichotomous outcome variable analogous to disease state, and normally distributed continuous predictors, we explored the error rate, coverage rate, bias and accuracy of various regression estimates. Our results indicate that weighted regression using RDS-II weights can lead to inflated type-I error, poor parameter coverage and biased results. When the goal of research is to estimate risk associated with exposure, we prefer Poisson regression to standard logistic regression because it directly estimates relative risk and at higher levels of outcome prevalence the odds ratio is a poor estimate of relative risk. Furthermore, our results show that at low prevalence Poisson regression performs well in terms of observed error rate, coverage and accuracy.

Several studies have reported using weighted regression (WR) techniques, with RDS-II weights, to account for the non-random nature of RDS samples [15, 36–40]. Results of this study indicated that weighted regression, to account for non-random sampling probability should not be undertaken for RDS data without careful consideration to the distribution of the weights used. The poor performance of weighted regression in this study can be attributed to the increased variability of the weighted regression estimates, as illustrated in Additional file 3: Figure S3 The weighted regression estimates are dependent on the reported network degree and a participant reporting very few connections in the community weighs heavily in the analysis and can act as a leverage point. The two most extreme simulated data sets from the population with prevalence of 10% and homophily of 1 are shown in Additional file 4: Figure S4. In this study, because population data were simulated and therefore completely known, reported network degree was equal to the actual network degree and participants were sampled based on their true degree of connectedness in the population. Despite perfect knowledge of network size, the presence of participants within the samples who reported very low degree (and hence had large weights) nevertheless unduly influenced the weighted regression estimates. That weighted regression performed poorly in these controlled circumstances should serve as a caution to future researchers. At the very least, unweighted estimates should always be reported. If weighted regression is performed care must be taken to investigate the influence of those assigned large weights and to perform sensitivity analysis on the degree information.

Our secondary analysis investigated populations where the outcome and network degree were correlated and largely replicated the findings of the primary investigation. When the outcome and degree are correlated, weighted regression results in inflated type-I error, except when those with the highest degree were in G1 (“diseased” group, outcome = 1). In this situation the error rate was virtually zero because those in G1 have the lowest RDS-II weights and so there are no leverage points that drive the high error rate in the other populations. This too though is undesirable because those in G2 (“healthy group”, outcome = 0) will tend to be leverage points and may nullify true relationships when they form a large majority of the population. Again, these findings suggest extreme caution using weighted regression with RDS samples.

We examined several techniques for dealing with clustering: GLM and GEE with data correlated within recruiter, seed or, both and with different covariance structures, as well as modelling the outcome value of the immediate recruiter as a model covariate. These results do not provide clear guidance on the best method of handling dependence in the data. None of the methods were consistently poor across models and populations. Including the outcome of a participant’s recruiter as a covariate may be a viable option; our results indicate that the extra parameter did not reduce the coverage rate and accuracy was actually minimally improved. We also note that in general, the impact of clustering on the variance of regression models is generally less than in the estimation of variance means or prevalence itself. For example, in the context of cluster randomized trials, Donner and Klar [41] discuss the decrease in variance in a regression model relative to a single mean or proportion. Nonetheless more work is necessary to determine the utility of this approach in populations where the relative activity depends on outcome group.

The performance of the unweighted GEE models was related to the working covariance structure and standard error adjustment used. Models fit with a compound-symmetric working covariance structure and any of the Classical, FIRORES, FIROEEQ or MBN adjustments to the standard error have acceptable overall error and coverage rates (models 19–23). However, slightly inflated error rates were observed for the population with prevalence of 50% and homophily of 1.5 and the population with prevalence 10% and no homophily. Coverage rates were generally close to 95% for these models. When an auto regressive term was used within seeds (models 27, 28), overall coverage dropped below 94%, this was also the case with a compound symmetric structure and no adjustment to the standard error (models 29, 30). The independent correlation structure (with no covariance between observations) performed poorly, with inflated type-I errors.

The glimmix procedure in SAS was used to model GEE with compound symmetric working covariance structures and various sandwich estimates (models 19–23). There were no appreciable differences in error rates, coverage rates or relative bias among the various standard error adjustments for these models. As shown in Additional file 6: Table S2 the glimmix models have slightly lower coverage rates, and inflated error rates for some populations, so we recommend simpler generalized linear models.

The accuracy of the models in terms of case prediction is higher for logistic regression than Poisson regression, although as can be seen in Fig. 3 the disparity is proportional to outcome prevalence. At lower prevalence levels, the Poisson model variance approaches the variance of the Binomial distribution and so model mis-specification decreases and accuracy increases.

Another method of simulating RDS data is through the use of exponential random graph models (ERGM). Spiller et al. [9] in their recent simulation study investigating the variability of RDS prevalence estimators, used ERGM to simulate multiple populations from distributions with specified homophily, prevalence, mean degree and relative activity. This approach creates networks that, when averaged over many simulations have the desired network parameters, though in practice individual populations will vary. In contrast, our approach randomly selected network degree from a specified distribution, and then randomly allocated group membership and ties in such a way as to achieve precise levels of prevalence and homophily. For each combination of desired network traits, a single population was created and multiple RDS samples were drawn, thereby allowing only a single source of variability, the RDS sampling process. Given that our research question of interest was how best to model data sampled using respondent-driven sampling from a networked population, we feel that fixing the population constant is the appropriate strategy, but examining the impact of the population simulation method is an area of future interest.

### Prevalence

Our findings are in line with other studies [9, 10, 42] that have found coverage rates substantially less than 95% in the estimation of prevalence from RDS samples. Our results also support using RDS-II over RDS-I. We found that the robust variance estimators of the *surveylogistic* procedure in SAS, using the RDS-II weights performed well (Table 3). One interesting finding is that, similar to the regression results, the weighted prevalence estimates are also susceptible to leverage points, but only at low prevalence (10%). When we more closely examined samples with large disparities in the outcome prevalence estimates we found that the disparity among estimators is caused entirely by individuals with low degree. The smallest reported network size in these samples was 2, in line with degree reported in the OHC study and in this simulation study, a reported degree of two is an accurate reflection of connectedness. The weights assigned to each participant are related not only to the participant’s reported degree but the distribution of degrees across the sample. If a sample contains a few reports of very large degree (as occurred in the OHC sample) then the weights allocated to those with lower reported degree will have greater impact. We found that prevalence estimators that incorporate weights are generally superior at moderate to high prevalence, but should be used with caution in samples with low outcome prevalence.

The appropriate use of weights in regression analysis is an area of active discussion. Our findings suggest that the use of weights is appropriate for determining population outcome prevalence, but not in the application of regression models for RDS samples. These results are in line with Lohr and Liu’s paper examining weighting in the context of the National Crime Victimization Survey [43]. In their survey of the literature they reported little debate surrounding the use of weights in the calculation of average population characteristics, but several competing views on the incorporation of weights into more complex analyses such as regression. More recent work by Miratrix et al. [44] further suggests that initial, exploratory analyses, as we are typically performing in RDS data should be performed without weights to increase power and that generalization to the entire population should be a secondary focus of subsequent samples.

In a simulation study the limitations stem from our own design. As an initial investigation into regression techniques and RDS data we chose to use complete data sets, so the effects of missing data are unknown. We also used a correctly-reported network degree, whereas in the OHC study we observed a tendency for people to report degree in clusters (such as 5, 10, 20, 100). Future work may focus more on log-link models, which seem promising. It would also be interesting to investigate what happens if the outcome responses are correlated with degree size, and, if better-connected people are better (or worse) off, a concern flagged by Reed et al. [45].

## Conclusion

Our results indicate that weighted regression should be used cautiously with RDS data. Unweighted estimates should always be reported, because weighted estimates may be biased and may not be valid in samples with a broad range of reported degree, such as the case with our motivating example of connectedness in an urban Indigenous population. Researchers are likely to have prior knowledge regarding the prevalence of the outcome in their target population (HIV prevalence, for instance), but much less likely to have knowledge regarding the homophily of the population. The greater the outcome prevalence, the greater the discrepancy between the odds ratio estimated from logistic regression and the relative risk. In light of this we suggest that a simple, unweighted, Poisson regression model is the most reliable method for modelling the likelihood of group membership from an RDS sample.

## Supplementary information

**Additional file 1: Figure S1.** Reported degree from the Our Health Counts Hamilton Study. The full range of reported degrees is shown in A, and a reduced range of degree < 125 is shown in B.

**Additional file 2: Figure S2.** Simulated degree used as the generating distribution for the simulated networked populations. The full range of reported degrees is shown in A, and a reduced range of degree < 125 is shown in B.

**Additional file 3: Figure S3.** Distribution of the odds ratio estimates from unweighted and weighted logistic regression models fit with the glm function in R (models 1 and 2). No adjustments were made for clustering.

**Additional file 4: Figure S4.** RDS-II weights from two samples drawn from population with 10% outcome prevalence (proportion in G1) and homophily of 1 that produced the smallest and largest weighted odds ratios. Top panels are members of G1, bottom panels are members of G2. The population OR and RR were 7.59 and 2.86, respectively. For Sample 1: unweighted OR = 3.2 weighted OR = 2.3, unweighted RR = 2.5, weighted RR = 2.0. For Sample 2: unweighted OR = 17.9, weighted OR = 73.7, unweighted RR = 4.2, unweighted RR = 4.1.

**Additional file 5: Table S1.** Observed type-I error rate for all models and simulated populations.

**Additional file 6: Table S2.** Observed risk parameter coverage rate for all models and simulated populations.

**Additional file 7: Table S3.** Bias with respect to the mean for all models and simulated populations.

**Additional file 8: Table S4.** Bias with respect to the median for all models and simulated populations.

**Additional file 9: Table S5.** Predictive accuracy across simulated populations for select models.

## Data Availability

The 12 simulated networked populations, as well as the complete list of sample identifiers for the populations with outcome prevalence = 10% are available on github: https://github.com/la189/NetworkedPopulations

## Abbreviations

GEE: Generalized estimating equation
GLM: Generalized linear model
GLMM: Generalized linear mixed model
RDS: Respondent-driven sampling
