# Supplementary figures and images for: Unweighted regression models perform better than weighted regression techniques for respondent-driven sampling data: results from a simulation study
Source: BMC Med Res Methodol. 2019 Oct 29;19:202. doi: 10.1186/s12874-019-0842-5 (PMC6819607; doi:10.1186/s12874-019-0842-5)

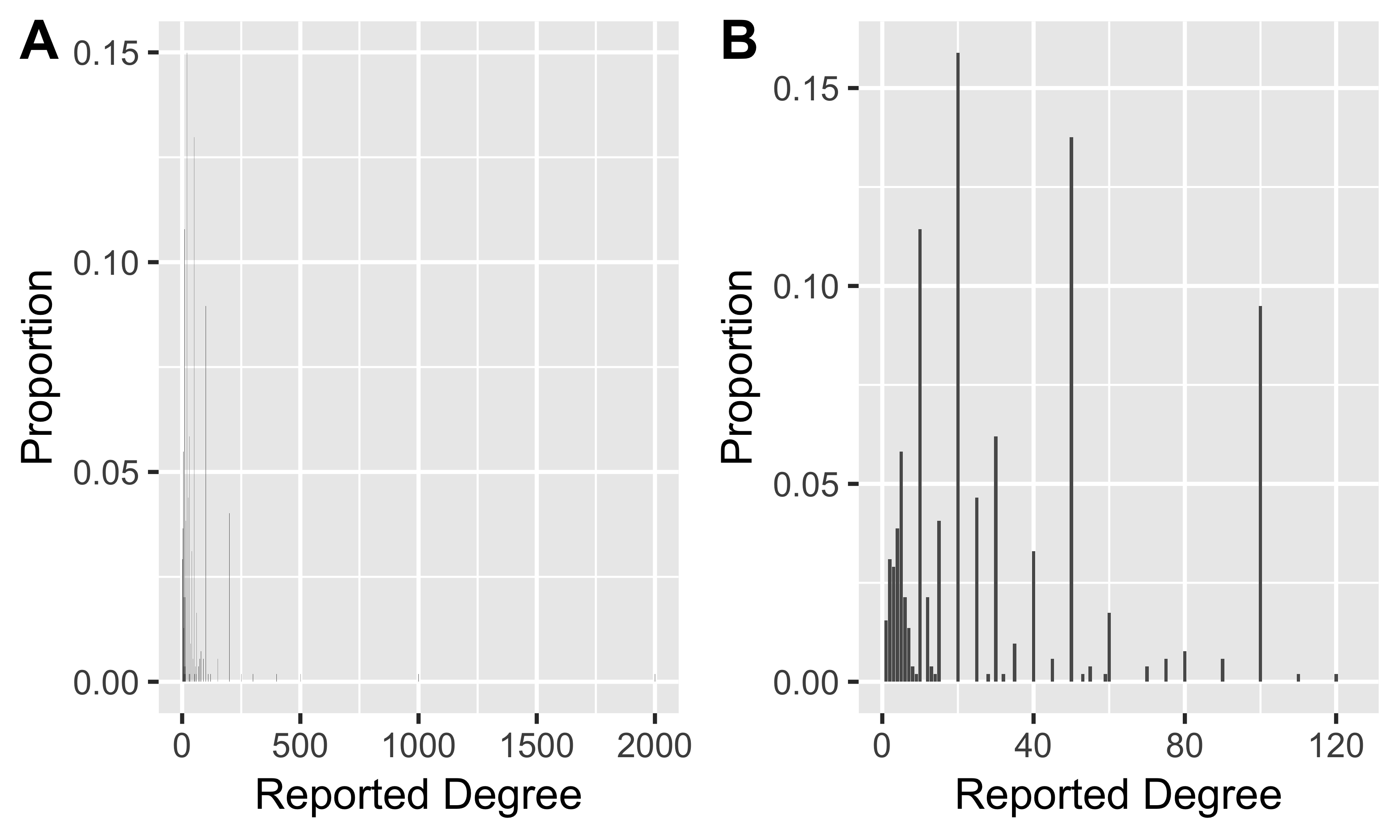

Supplement: Supplementary file 1 — Additional file 1: Figure S1. Reported degree from the Our Health Counts Hamilton Study. The full range of reported degrees is shown in A, and a reduced range of degree < 125 is shown in B. [file 12874_2019_842_MOESM1_ESM.png]

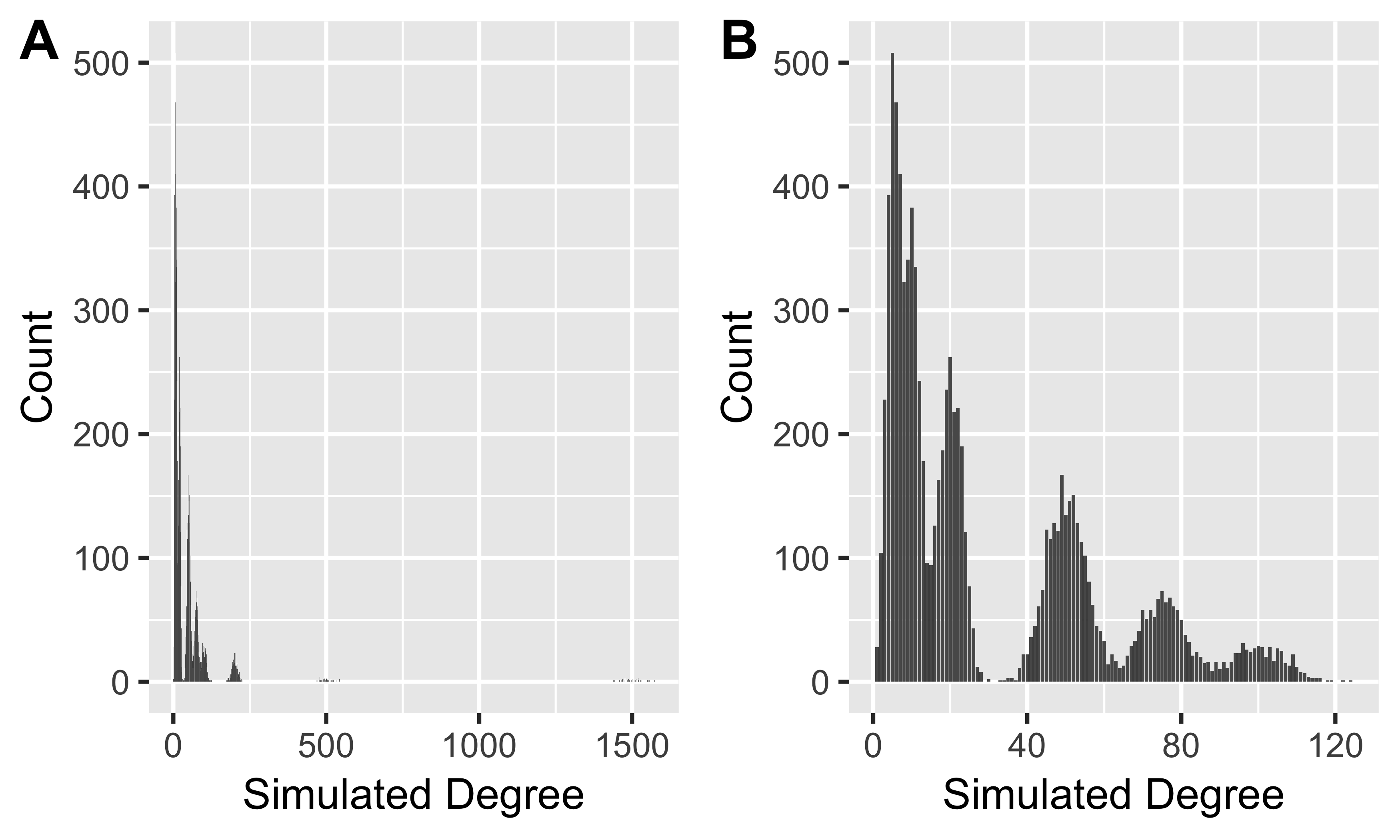

Supplement: Supplementary file 2 — Additional file 2: Figure S2. Simulated degree used as the generating distribution for the simulated networked populations. The full range of reported degrees is shown in A, and a reduced range of degree < 125 is shown in B. [file 12874_2019_842_MOESM2_ESM.png]

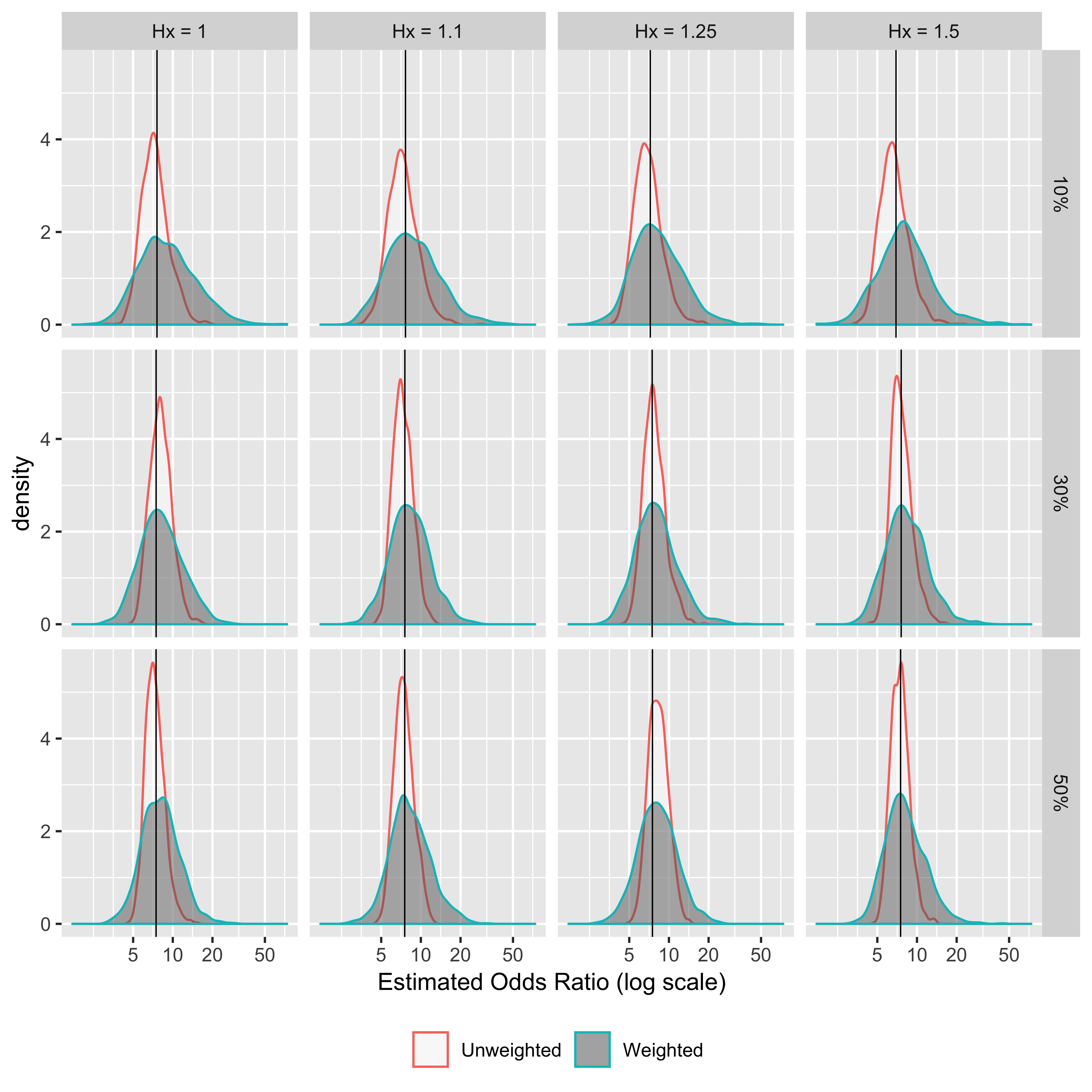

Supplement: Supplementary file 3 — Additional file 3: Figure S3. Distribution of the odds ratio estimates from unweighted and weighted logistic regression models fit with the glm function in R (models 1 and 2). No adjustments were made for clustering. [file 12874_2019_842_MOESM3_ESM.png]

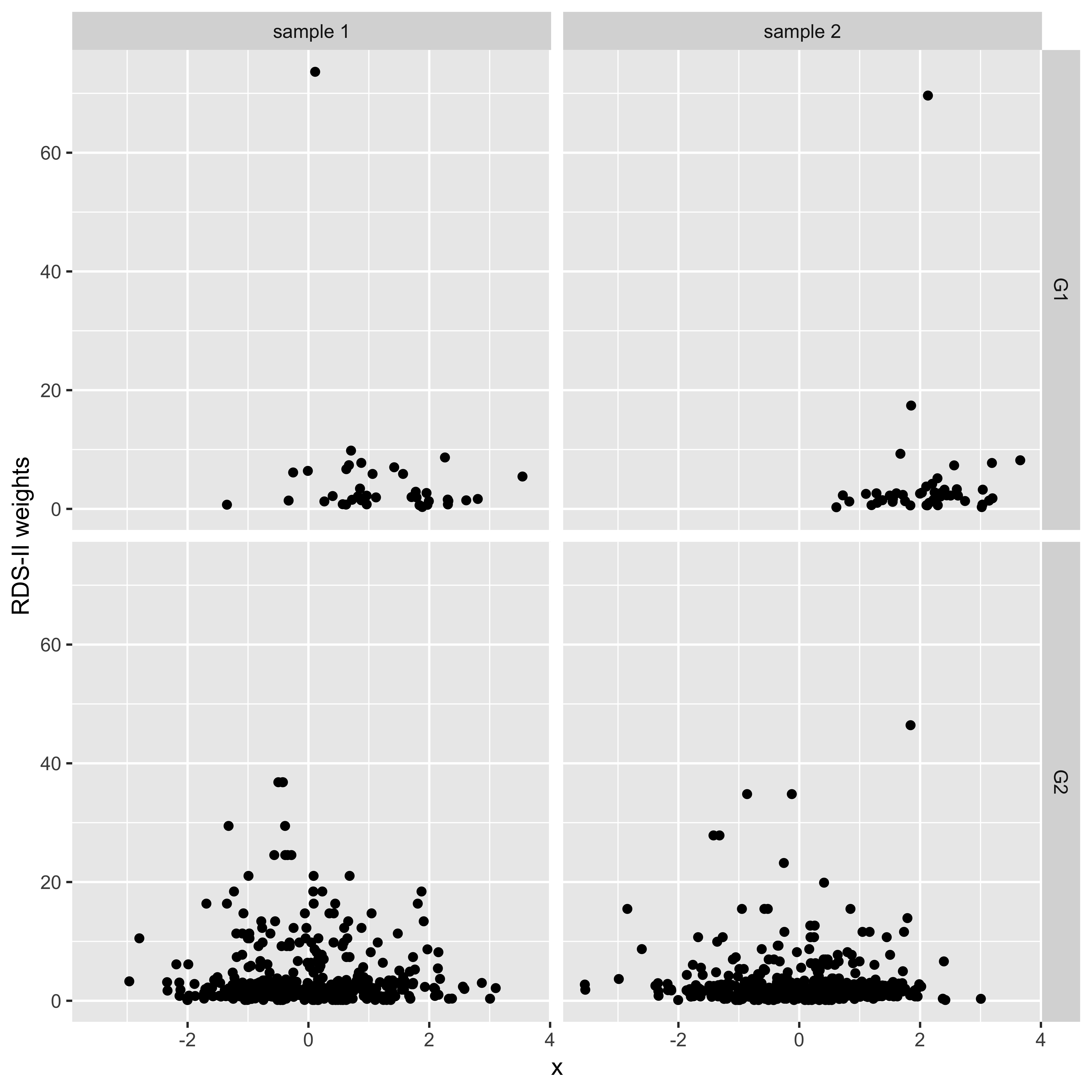

Supplement: Supplementary file 4 — Additional file 4: Figure S4. RDS-II weights from two samples drawn from population with 10% outcome prevalence (proportion in G1) and homophily of 1 that produced the smallest and largest weighted odds ratios. Top panels are members of G1, bottom panels are members of G2. The population OR and RR were 7.59 and 2.86, respectively. For Sample 1: unweighted OR = 3.2 weighted OR = 2.3, unweighted RR = 2.5, weighted RR = 2.0. For Sample 2: unweighted OR = 17.9, weighted OR = 73.7, unweighted RR = 4.2, unweighted RR = 4.1. [file 12874_2019_842_MOESM4_ESM.png]
